# Correlation between insulin-based and C-peptide based homeostatic model assessment of insulin resistance in adults without diabetes in a sub-Saharan African setting: a cross-sectional study

**DOI:** 10.1186/s13104-022-06214-w

**Published:** 2022-10-12

**Authors:** Blessing N. Tekoh, Esther Astrid E. Mbono-Samba, Martine Claude Etoa-Etoga, Manuela Audrey Eko, Falmata Amazia, Batakeh Ba Agoons, Jobert Richie Nansseu, Jean Joel Bigna, Vicky Jocelyne Ama-Moor

**Affiliations:** 1 Department of Biochemistry, Faculty of Medicine and Biomedical Sciences, University of Yaoundé 1, Yaoundé, Cameroon; 2 Department of Clinical Sciences, Faculty of Medicine and Pharmaceutical Sciences, University of Douala, Douala, Cameroon; 3 Department of Internal Medicine and Specialities, Faculty of Medicine and Biomedical Sciences, University of Yaoundé 1, Yaoundé, Cameroon; 4 Department of Public Health, Faculty of Medicine and Biomedical Sciences, University of Yaoundé 1, Yaoundé, Cameroon; 5 Department of Epidemiology and Public Health, Centre Pasteur of Cameroon, Yaoundé, P.O. 1274, Cameroon; 6 Department of Biochemistry, Yaoundé University Teaching Hospital, Yaoundé, Cameroon

**Keywords:** HOMA-IR, Insulin, C-peptide, Insulin resistance, Sub-saharan Africa.

## Abstract

**Objective:**

To assess the correlation between the insulin-based and C-peptide based HOMA-IR in the general population without diabetes in sub-Saharan Africa as well as to identify factors associated with IR.

**Results:**

This was a cross-sectional study in urban settings in Yaoundé, Cameroon. We included 84 people with a body mass index (BMI) ≥ 18.5 Kg/m² and without diabetes (females: 72.6%; mean age: 37 years). IR was assessed using the following formulae: HOMA-IR_INS_ = fasting insulin (mU/ml) x fasting plasma glucose (FPG) (mmol/L)/ 22.5; HOMA-IR_CP1_ = fasting C-peptide (mU/ml) x FPG (mmol/L)/ 22.5; and HOMA-IR_CP2_ = 1.5 + (FPG (mg/dl) x fasting C-peptide (ng/ml))/ 2800. Correlation (rho) between HOMA-IR_INS_ and C-peptide based HOMA-IR was investigated using the Spearman rank test. The median (25th -75th percentiles) HOMA-IR_INS_, HOMA-IR_CP1_, and HOMA-IR_CP2_ were: 1.94 (1.36–3.50), 0.18 (0.11–0.27) and 9.91 (6.81–14.52), respectively. There was no correlation between the insulin-based and C-peptide-based HOMA-IR indices: rho = 0.043, *p* = 0.697. IR (HOMA-IR_INS_ ≥ 2.8) was associated with obesity: A BMI ≥ 30 Kg/m² (adjusted odds ratio (aOR): 16.9, 95% confidence intervals (CI): 3.1–92.5) and being a student (aOR: 8.9, 95%CI: 2.1–38.2) were associated with IR.

**Supplementary Information:**

The online version contains supplementary material available at 10.1186/s13104-022-06214-w.

## Introduction

Insulin resistance (IR) is defined clinically as the inability of a known quantity of exogenous or endogenous insulin to increase glucose uptake and utilization in an individual as much as it does in a normal population [1]. There are various methods for the assessment of insulin resistance. The reference one is the hyperinsulinemic euglycemic clamp technique because it reflects the total body glucose metabolism. However, it is not used in day-to-day clinical practice because it is costly, labour-intensive, and cumbersome to perform [2]. Notwithstanding, several simpler methods to assess IR are available. The most widely used in clinical practice and research is the homeostatic model assessment (HOMA) of IR index. Indeed, the HOMA-IR is easily calculated and shows a significant correlation with the clamp technique [3].

C-peptide, a marker of endogenous insulin secretion, is also used to evaluate IR. Actually, C-peptide is secreted in equimolar concentrations as insulin, assayed under the same pre-analytic conditions, doesn’t undergo the first pass hepatic metabolism and has a longer half-life than insulin [4]. The cheaper cost of assaying C-peptide too is an added advantage. Both insulin and C-peptide levels are used by researchers and in clinical practice for the calculation of the HOMA-IR index and at times interchangeably. However, the agreement between these different ways of obtaining the HOMA-IR index has not been fully explored, especially in populations living in sub-Saharan Africa (SSA).

Hence in this study, we assessed the correlation between the insulin-based HOMA-IR index and the C-peptide based HOMA-IR index in a group of people without diabetes living in a SSA urban setting. In addition, we determined the prevalence of IR in the study population as well as associated factors.

## Methods

### Study design, setting, and population

In this cross-sectional study, participants were included from February to March 2021 in the city of Yaoundé, Cameroon. Yaoundé is an urban city and the capital of Cameroon, a lower-middle income country located in Central Africa. This study included participants enrolled both from the hospital and community: volunteers without a known history of diabetes were recruited at the outpatient unit of the Yaoundé Central Hospital and through health campaigns in churches. Both male and female subjects aged at least 21 years, with a body mass index (BMI) ≥ 18.5 Kg/m^2^ and willing to participate in the study were included. Heavy smokers, subjects with diabetes (fasting plasma glucose ≥ 1.26mg/dl), those with other documented chronic diseases, those who experienced recent episodes of fever or were on medications affecting glucose/lipid metabolism, and pregnant or breastfeeding women were excluded.

The minimum required sample size was estimated at 82 participants, considering a power of 90% and a two-sided test with 0.05 as level of signification. We considered a correlation of zero between the insulin-based HOMA-IR index and the C-peptide based index under the null hypothesis. For the alternative hypothesis, we considered a difference of 0.35.

All participants included in this study gave a written consent. The protocol was approved by the Ethics Committee for Human Health Research for the Centre Region of Cameroon (N° 1127/CRERHSC/2020). The study was performed in accordance with the Declaration of Helsinki and all participants gave informed consent.

### Data collection and variables

Subjects underwent an interview during which socio-demographic data and medical history were collected. Subsequently, a physical examination including blood pressure and anthropometric measurements was performed. The BMI was calculated and participants grouped into three categories: normal weight (18.5–24.9 Kg/m²), overweight (25.0–29.9) or obesity (≥ 30.0) [5]. The waist circumference (WC) was measured with a flexible inelastic tape and central obesity was defined as a WC ≥ 94cm in males and ≥ 80cm in females [6].

With full aseptic precautions, fasting blood samples were collected after an overnight fasting of at least 8h. All samples collected were transported to the biochemistry laboratory of the Yaoundé University Teaching Hospital within 4h for centrifugation and further analysis. Fasting plasma glucose was assayed daily using the colorimetric method with Biolabo kits. For insulin and C-peptide assays, centrifuged blood sample were stored at -20°C until analysed. The Immuno-Biological Laboratories international kits were used to assay fasting insulin and C-peptide levels using the sandwich ELISA method.

IR was assessed using the following formulae:

- HOMA-IR_INS_: fasting insulin (mU/ml) x FPG (mmol/L) / 22.5 [3];
- HOMA-IR_CP1_: fasting C-peptide (mU/ml) x FPG (mmol/L) / 22.5 [7];
- HOMA-IR_CP2_: 1.5 + (FPG (mg/dl) x fasting C-peptide (ng/ml)) / 2800 [8].

The insulin-based HOMA-IR was considered as the reference method to assess insulin resistance. IR was defined as a HOMA-IR_INS_ index ≥ 2.8 as used in a non-diabetic population in Bangladesh, as there is yet no well agreed recommended cut-off values in Cameroon [9].

### Statistical analysis

Data were entered and coded using the software SPSS version 24.0 (IBM Corp. Released 2016. IBM SPSS Statistics for Windows, Version 24.0. Armonk, NY: IBM Corp.) and subsequently analysed using R version 4.1.1. Data were presented as count (percentage) for categorical variables; mean ± standard deviation (SD) or median (25th − 75th percentiles) for quantitative variables after checking for normality. Using the *glm* function, a logistic regression was used to investigate the association between IR (dependent variable) and socio-demographic and clinical parameters (independent variables). Eligible variables with a p value < 0.25 in the univariable model were selected for the multivariable model. The final multivariable model was the one with the lowest Akaike Information Criterion (AIC). The strength of association was measured with odds ratios (OR) and their 95% confidence intervals (CI). The correlation between surrogate estimates was assessed using the Spearman rank test providing the rho (ρ) coefficient of correlation. A p-value < 0.05 was considered statistically significant.

## Results

### Characteristics of the study population

One hundred and ten people responded to our invitation to participate in the study, and after assessment for eligible criteria, 84 were included in final analyses (Supplementary file 1 Figure S1). The mean age was 37.0 ± 13.3 years. Most participants were women, were single, had a higher level of education, and were Semi-Bantu by ethnicity (Table 1). Table 2 presents the medical history as well as clinical and biological characteristics of the study population. Majority of participants had obesity (Table 2).

**Table 1:** Sociodemographic characteristics of the study population

Variables	n (%)
Sex	
- Female	61 (72.6)
- Male	23 (27.4)
Occupation	
- Liberal (no salary)	32 (38.1)
- Student	24 (28.6)
- Non liberal (on salary)	16 (19.0)
- Housewife	6 (7.1)
- Retired	3 (3.6)
- Jobless	3 (3.6)
Marital status	
- Single	44 (52.4)
- Married	34 (40.5)
- Widow	5 (6.0)
- Divorced	1 (1.2)
Level of education	
- Higher	45 (53.6)
- Secondary	26 (31.0)
- Primary	12 (14.3)
- None	1 (1.2)
Ethnicity	
- Semi-Bantu	61(72.6)
- Bantu	20 (23.8)
- Sudanese	3 (3.6)

**Table 2:** Medical history, clinical, and biological parameters

Variables	N = 84
First degree relative with diabetes	28 (33.3)
Alcohol consumption	29 (34.5)
Tobacco consumption	4 (4.8)
Body mass index, kg/m^2^	28.4 ± 5.7
Body mass index class	
- Normal	28 (33.3)
- Overweight	25 (29.8)
- Obesity	31 (36.9)
Systolic blood pressure (mmHg)	122.1 ± 10.4
Diastolic blood pressure (mmHg)	78.1 ± 7.5
Fasting plasma glucose (mmol/l)	4.9 (4.6–5.3)
C-peptide (ng/ml)	264.9 (163.8–397.3)
Insulin (mUI/l)	9.37 (6.00–14.18)
HOMA-IR_INS_	1.94 (1.36–3.50)
HOMA-IR_CP1_	0.18 (0.11–0.27)
HOMA-IR_CP2_	9.91 (6.81–14.52)

Data are n (%), mean ± standard deviation, or median (25th -75th percentiles)

HOMA: Homeostasis model assessment, Ins: insulin, CP: C-peptide

### Correlation between insulin-based and C-peptide HOMA-IR

The coefficient of correlation between HOMA-IR_INS_ and HOMA-IR_CP1_ and between HOMA-IR_INS_ and HOMA-IR_CP2_ was the same: ρ = 0.043, p = 0.697. Supplementary file 2 Figure S2 and Supplementary file 3 Figure S3 present the distribution of C-peptide based HOMA-IR according to HOMA-IR_INS_.

### Factors associated with insulin resistance

In all, 31 (36.9%) participants were found with IR. In univariable analysis, obesity (OR: 4.9; 95% CI: 1.5–16.2) and central obesity (OR: 3.7; 95% CI: 1.3–10.5) were significantly associated with IR (Table 3). In the multivariable model, obesity (aOR: 16.9, 95% CI: 3.1–92.5) and being a student (aOR: 8.9, 95% CI: 2.1–38.2) were the only factors independently associated with IR (Table 3). In an exploratory model including both occupation and central obesity (replacing obesity by central obesity), IR was significantly associated with central obesity: aOR: 8.3 (95% CI: 2.3–41.0).

**Table 3:** Univariable and multivariable analysis of factors associated with insulin resistance

Variables	IR (+) N = 31	IR (-) N = 53	Univariable model		Multivariable model	
			**Odds ratio (95% CI)**	**p value**	**Adjusted odds ratio (95% CI)**	**p value**
**Age**						
- < 45 years	17 (54.8)	26 (49.1)	1			
- ≥ 45 years	14 (45.2)	27 (50.9)	0.97 (0.34–2.63)	0.951		
**Sex**						
- Female	22 (71.0)	39 (73.6)	1			
- Male	9 (29.0)	14 (26.4)	1.14 (0.43–3.06)	0.795		
**Profession**						
- Active	15 (48.8)	33 (62.3)	1		1	
- Student	13 (41.9)	11 (20.8)	2.60 (0.95–7.13)	0.063	8.91 (2.08–38.17)	0.003
- Housewife	2 (6.5)	4 (7.5)	1.10 (0.18–6.68)	0.918	0.63 (0.09–4.38)	0.643
- Retired	1 (3.2)	2 (3.8)	1.10 (0.09–13.09)	0.940	1.59 (0.10–26.30)	0.747
- Jobless	0 (0.0)	3 (5.7)	NE		NE	
**Marital status**						
- Single	16 (51.6)	28 (52.8)	1			
- Married	14 (45.2)	20 (37.7)	1.23 (0.49–3.07)	0.665		
- Widow	1 (3.2)	4 (7.5)	0.48 (0.05–4.26)	0.476		
- Divorced	0 (0.0)	1 (1.9)	NE	NE		
**Level of education**						
- Secondary or higher	26 (83.9)	45 (84.9)	1			
- None or primary	5 (16.1)	8 (15.1)	1.08 (0.32–3.65)	0.899		
**Ethnicity**						
- Bantu	7 (22.6)	13 (24.5)	1			
- Semi-Bantu	23 (74.2)	38 (71.7)	1.12 (0.39–3.23)	0.828		
- Sudanese	1 (3.2)	2 (3.8)	0.93 (0.07–12.14)	0.955		
**First degree relative with diabetes**	12 (38.7)	16 (30.2)	1.46 (0.58–3.70)	0.425		
**Alcohol consumption**	10 (32.3)	19 (35.8)	0.85 (0.33–2.18)	0.083		
**Tobacco consumption**	0 (0.0)	4 (7.5)	NE			
**BMI class**						
- Normal	5 (16.1)	23 (43.4)	1		1	
- Overweight	10 (32.3)	15 (28.3)	3.07 (0.87–10.76)	0.080	4.35 (0.999-19.0)	0.050
- Obesity	16 (51.6)	15 (28.3)	4.91 (1.48–16.23)	0.009	16.93 (3.10-92.54)	0.001
**Central obesity**	25 (80.6)	28 (52.8)	3.72 (1.31–10.54)	0.013		
**SBP ≥ 130 mmHg**	9 (29.0)	13 (24.5)	1.26 (0.45–3.40)	0.651		
**DBP ≥ 85 mmHg**	6 (19.4)	14 (26.4)	0.67 (0.21–1.91)	0.465		

IR: insulin resistance, OR: odds ratio, CI: confidence interval, NE: not estimable, BMI: body mass index, SBP: systolic blood pressure, DBP: diastolic blood pressure

## Discussion

In this study of non-diabetic individuals, more than one-third were identified with IR. This estimate is higher than reports from Benin and South Africa (all located in SSA): 15.4% and 25.5%, respectively [10, 11]. This difference can be attributed to the use of higher cut-off values (respectively 3.2 and 3.1 in these studies vs. 2.8 in the present one). The estimate obtained in this study was lower compared to the one found in a non-diabetic population in Bangladesh (52.4%) using the same surrogate index and cut-off to define IR [9]. This could be explained by the high proportion of obesity (48.5%) in their study population as well as genetic factors. Indeed, numerous studies have suggested different effects of IR across racial/ethnic groups. Postulated mechanisms are: varying basal insulin secretion levels, distribution of adiposity, genetics, environmental factors, and psychosocial stress [12–14].

IR was associated both with central obesity and general obesity in univariable analysis; after multivariable analysis in the main analysis, general obesity and being a student were the factors independently associated with IR. Moreover, the exploratory multivariable analysis showed an association between central obesity and IR. As such, this study confirms the well-established association between IR and central obesity and general obesity [1, 15]. Although central obesity was not associated with IR in the main analysis after multivariable analysis including obesity, more than half (52.8%) of insulin sensitive participants had central obesity. A similar trend of a relatively high prevalence of central obesity in non IR subjects was observed, especially among women [11]. This could be due to the fact that Africans have less visceral fat which is mainly incriminated with increased cardio-metabolic risk and thus raises again the issue of the need for specific waist circumference cut-offs for sub-Saharan Africans [16].

Findings from this study also suggest that being a student might be associated with IR. This could be explained by lifestyle habits common to this group (hypercaloric diets, altered sleep patterns) which are associated with a decrease in insulin sensitivity [17]. In addition, most participants in this group (87.5%) pursued studies in the medical field with night shifts. Indeed, a study demonstrated that shift working could be a risk factor for developing IR when comparing the metabolic profiles of healthcare shift workers to their non-shift counterparts [18].

There was no correlation between insulin-based HOMA-IR and C-peptide based HOMA-IR. This is contrary to findings from India that reported a significantly positive linear correlation in type 2 diabetes mellitus (T2DM) patients, even though it was weak [19]. This disparity could be explained by numerous factors, the difference in insulin assay methods being one of them. In this study from India [19], insulin was assayed using an automated biochemistry analyser based on electrochemiluminescence, meanwhile in our study, insulin was assayed using the ELISA sandwich principle. It is suggested that different assay methods/kits may show significant variations in insulin levels [20]. Also, the two studies were carried out on different populations. Variations in glucose and insulin levels are influenced by the degree of glucose tolerance and the use of medications; thus, their relationship in T2DM patients may not reflect systemic insulin sensitivity. Fasting IR indices are therefore less reliable in T2DM patients [13, 20]. C-peptide is a by-product of insulin synthesis and even though they are secreted in equimolar quantities, they have different molar masses. C-peptide was long considered not to have a biological activity, hence has no available conversion to international units (IU). Researchers have used the conversion unit for insulin to transform C-peptide values into IU since they are secreted in an equimolar fashion, but we didn’t find a correlation between insulin and C-peptide HOMA-IR values using these two formulas. This raises the issue of interchanging insulin with C-peptide in this formula although cautious interpretation is needed.

Nonetheless, several studies have identified C-peptide to be a biomarker of IR and have developed formulae based on its fasting levels to evaluate insulin sensitivity [8, 21, 22]. One of these is the modified C-peptide HOMA-IR formula (HOMA_CP2_) which correlated significantly with insulin HOMA-IR values (r = 0.689, p < 0.0001) when it was developed in 21 healthy subjects of the Chinese population [8]. This mismatch may be due to racial differences, as it is reported that the performance of surrogate indices may depend on the race [13, 23]. Also, insulin in our study was assayed on a single blood fasting sample meanwhile in this Chinese population, insulin levels were assayed after an oral glucose tolerance test which is superior to fasting samples [8].

## Limitations

Nevertheless, this study should be interpreted considering some limitations, one of which is the use of the HOMA-IR index as gold standard method to assess IR instead of the euglycaemic-hyperinsulinaemic clamp. Additionally, insulin levels used were assayed from a single blood sample instead of the mean from three samples obtained at a five-minutes interval. Moreover, participants were declared non-diabetic from a single fasting blood glucose assay. Despite these limitations, this study is among the few studies which have investigated the correlation between the C-peptide-based indices of IR and another surrogate index in SSA.

## Electronic supplementary material

Below is the link to the electronic supplementary material.

Supplementary Material 1

Supplementary Material 2

Supplementary Material 3

## Data Availability

Data that support the findings of this study are available upon request addressed to the corresponding author.

## List of abbreviations

BMI: body mass index.
CI: confidence interval.
DBP: diastolic blood pressure.
FPG: fasting plasma glucose.
HOMA: homeostatic model assessment.
IR: insuline resistance.
OR: odds ratio.
SBP: systolic blood pressure.
SSA: sub-Saharan Africa.
