# Supplementary figures and images for: Correlation between insulin-based and C-peptide based homeostatic model assessment of insulin resistance in adults without diabetes in a sub-Saharan African setting: a cross-sectional study
Source: BMC Res Notes. 2022 Oct 12;15:322. doi: 10.1186/s13104-022-06214-w (PMC9558417; doi:10.1186/s13104-022-06214-w)

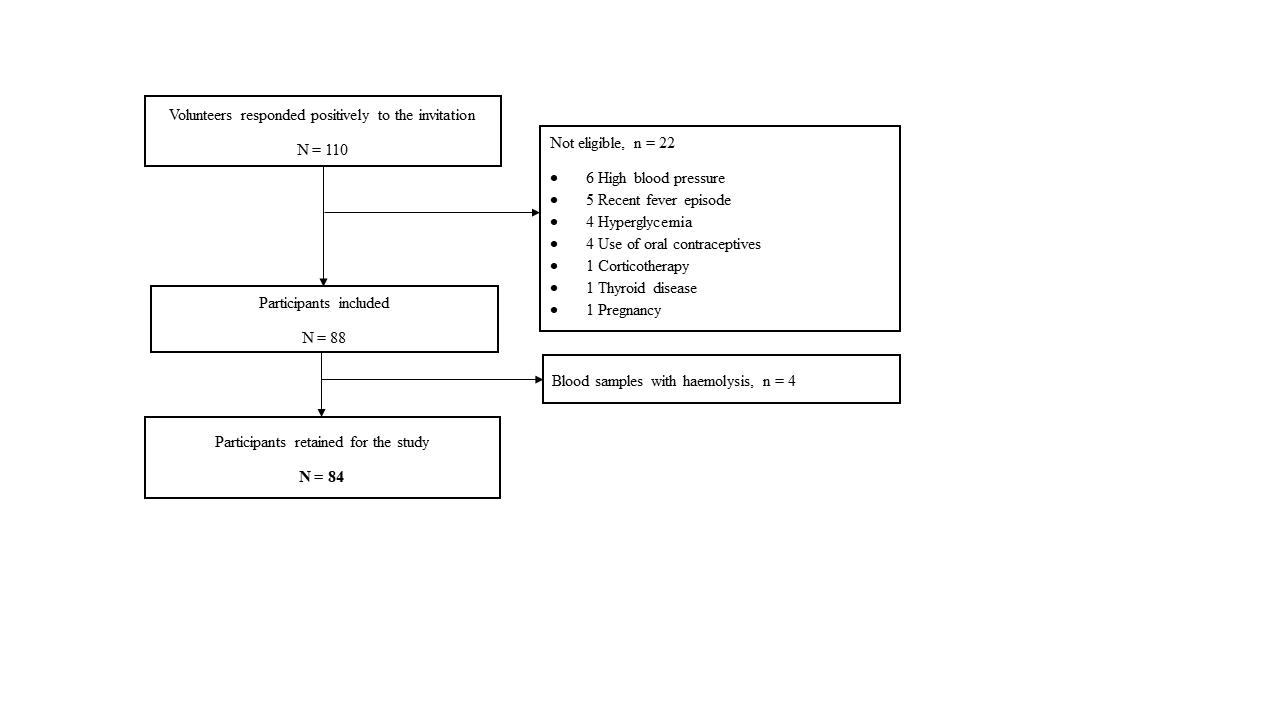

Supplement: Supplementary file 1 — Supplementary Material 1 [file 13104_2022_6214_MOESM1_ESM.png]

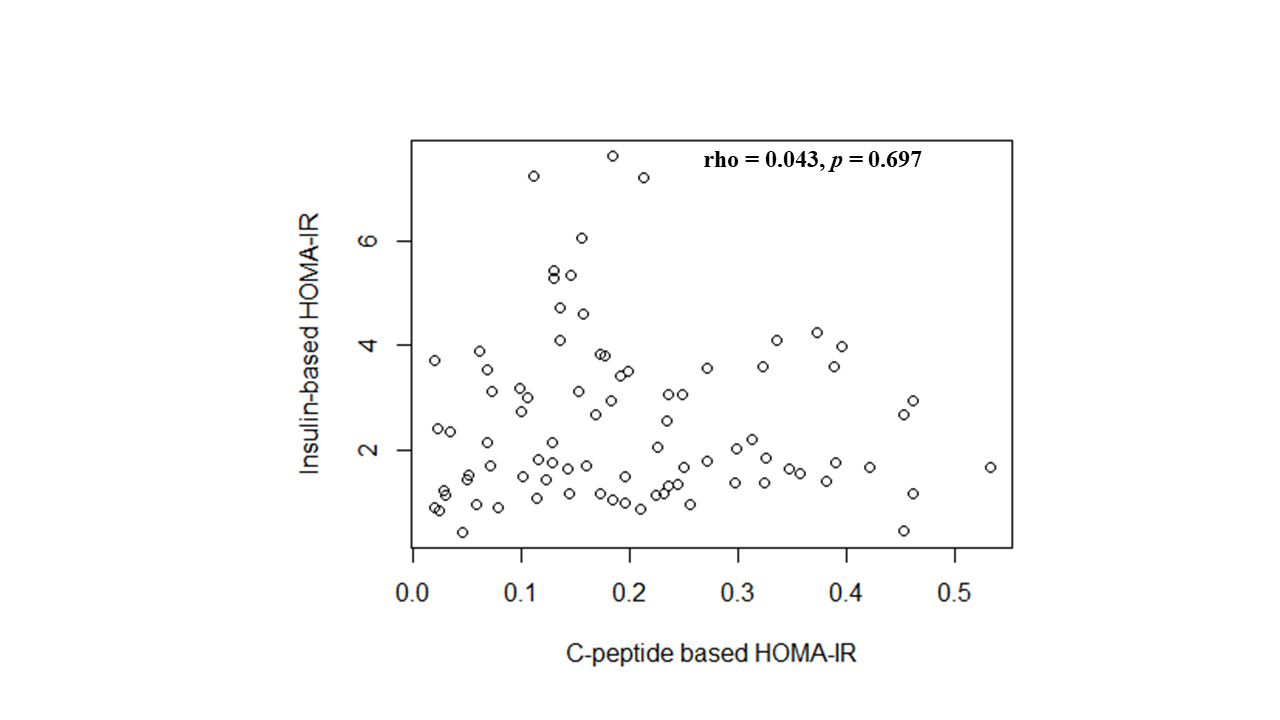

Supplement: Supplementary file 2 — Supplementary Material 2 [file 13104_2022_6214_MOESM2_ESM.png]

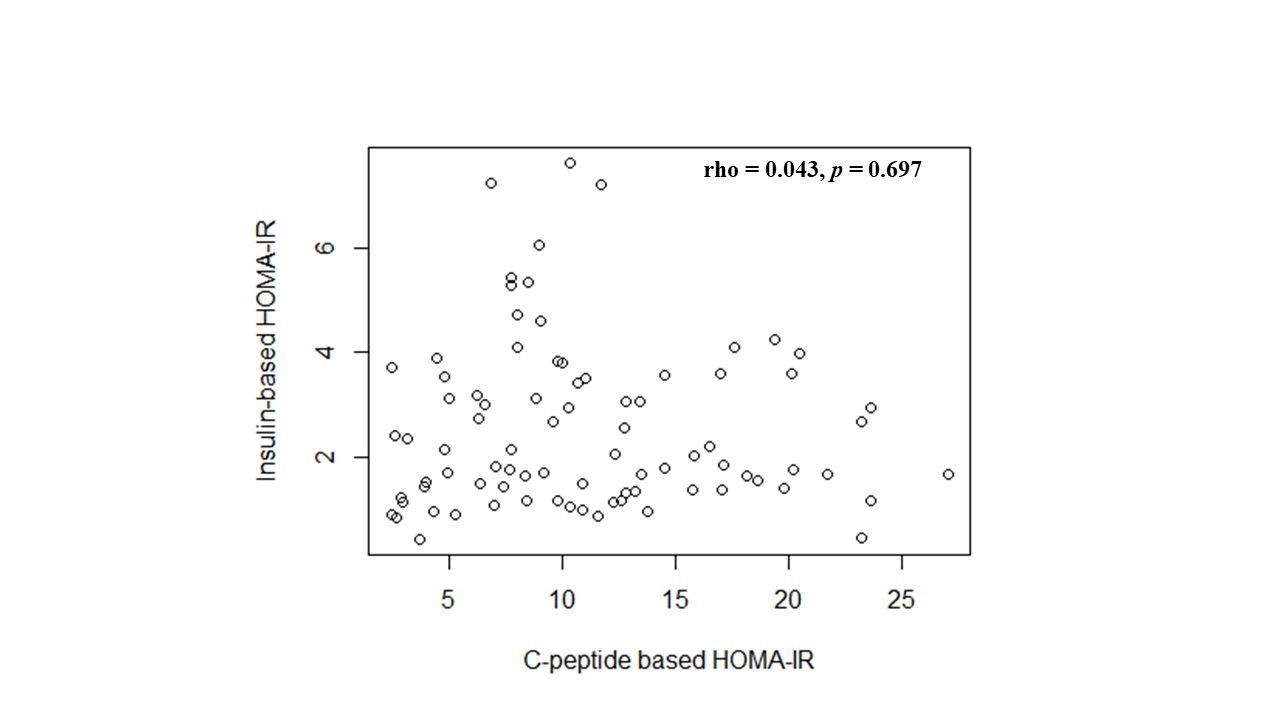

Supplement: Supplementary file 3 — Supplementary Material 3 [file 13104_2022_6214_MOESM3_ESM.png]
